# Imaging manifestations of ductal adenoma of the breast: A case report

**DOI:** 10.1515/biol-2022-0917

**Published:** 2024-07-16

**Authors:** Yaning Zhu, Haitong Yu, Zhaolong Zheng, Zewen Liu, Qin Li, Xiqing Wu

**Affiliations:** Department of Radiology, WeiFang Traditional Chinese Hospital, No. 1055 Weizhou Road, Weifang 261041, China; Medical Imaging Department, Weifang Medical University, Weifang 261053, Shandong, People’s Republic of China

**Keywords:** tubular adenoma, breast cancer, magnetic resonance imaging, ultrasound

## Abstract

Tubular adenomas of the breast are rare benign epithelium-derived tumours, and so few cases have been reported. Most often, the tumours are palpable, well-circumscribed masses in women of childbearing age and are commonly diagnosed as fibroadenomas both clinically and radiographically. We describe the case of a premenopausal patient with tubular adenoma of the breast who presented with small nipple discharge and a palpable breast mass. On imaging, tubular adenomas are practically indistinguishable from fibroadenomas and most commonly present as oval, circumscribed masses that are hypoechoic on ultrasound. On magnetic resonance imaging (MRI), tubular adenomas may present as lobulated or oval masses with a hyperintense signal on T2-weighted imaging and inhomogeneous internal enhancement on dynamic contrast-enhanced MRI. Pathologic findings after resection of the mass confirmed the diagnosis of tubular adenoma.

## 1 Background

Tubular adenoma of the breast is a clinically rare, benign epithelium-derived tumour that accounts for approximately 0.13–1.70% of benign breast tumours [1]. Typically, tubular adenomas occur in young women of childbearing age and rarely occur before menarche or after menopause [2]. The classical histological characteristic of tubular adenoma is the proliferation of packed tubular structures within a small amount of fibrous stroma. Tubular adenomas present clinically painless, palpable nodules [3]. The final diagnosis of tubular adenoma of the breast requires histopathological analysis [1]. Due to its low incidence, most of the reports in the literature describe only clinicopathological features. There are few reports on the usefulness of radiologic and pathologic methods for identifying tubular adenoma. Breast ultrasound is an important means of screening and diagnosing breast diseases [4]. Magnetic resonance imaging (MRI) has a high resolution and can provide a large amount of detailed anatomical and histologic information. Radiologists and clinicians often have a poor understanding of tubular adenoma, which is easily misdiagnosed as fibroadenoma before surgery [5]. Both ultrasound and MRI provide a reliable basis for the diagnosis of benign lesions.

In this study, the imaging, pathological, and clinical data of a patient with tubular adenoma confirmed by surgery and pathology at our hospital were retrospectively analysed to improve the ability of imaging doctors to recognize breast tubular adenoma images as well as the preoperative diagnosis rate.

## 2 Case presentation

**Figure 1:**
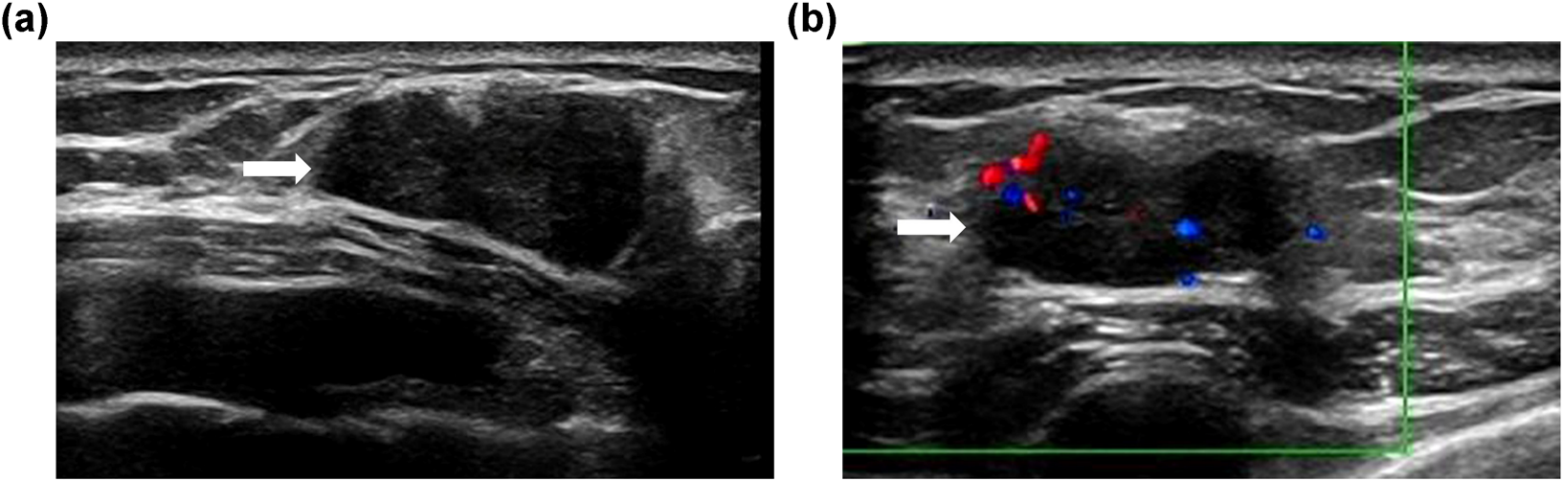
Ultrasound exam of her left breast revealed oval, parallel, circumscribed, hypoechoic masses (arrows) with internal vascularity (a and b).

A 43-year-old female was found to have a painless mass in her left breast 2 years prior that was not treated. Specialist examination revealed that the appearance of both breasts was normal, lacking an orange peel appearance or the dimple sign. The left nipple was accompanied by a small amount of discharge. A mass of approximately 20 mm × 20 mm was palpable in the upper quadrant of the left inner mammary gland. Ultrasound revealed a glandular layer in the left outer lower breast quadrant and a nodular hypoechoic lesion 2.1 cm × 1.4 cm in size with a clear boundary and lobed shape. Colour Doppler ultrasound revealed a blood flow signal in the lesion. The breast imaging reporting and data system classification was 4b (Figure 1a and b).

**Figure 2:**
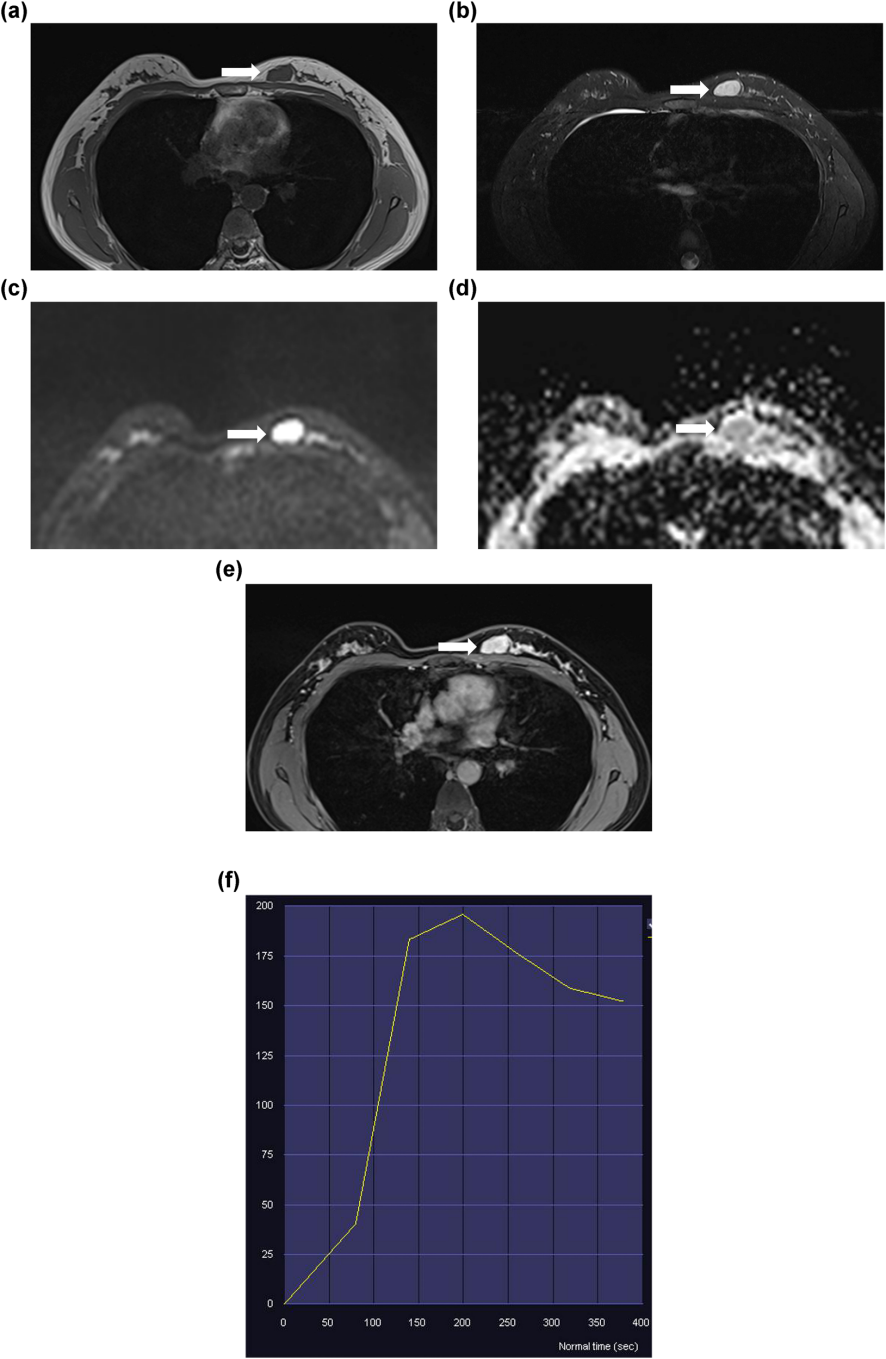
MRI of lesions of the left breast (arrows). (a) Lesions with Low signal intensity on T1-weighted images. (b) High signal intensity on T2-weighted images. (c and d) High apparent diffusion coefficient value. (e and f) Dynamic contrast-enhanced imaging with time signal intensity curves for washout pattern.

**Figure 3:**
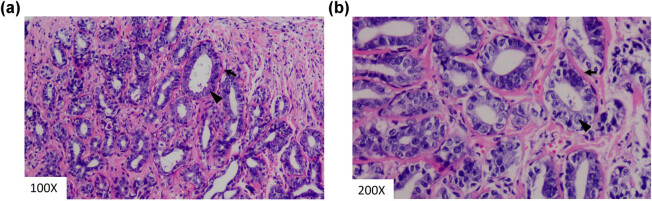
Tubular adenoma (a and b). Hematoxylin and eosin of core needle biopsy shows a lesion with well-defined borders consisting of small, lined by a basal layer of myoepithelial cells (arrow) and overlying glandular epithelium (arrowhead). There is minimal intertubular fibrous tissue.


The patient underwent contrast-enhanced breast MRI, which revealed a lobulated mass in the left breast, presenting with a hypointense signal on T1-weighted imaging (Figure 2a) and a hyperintense signal on T2-weighted imaging (Figure 2b). The lesion showed an elevated signal with a high apparent diffusion coefficient (1.41 × 10^−3^ mm^2^/s) on diffusion-weighted imaging (Figure 2c and d). Dynamic contrast-enhanced MRI revealed that the mass had inhomogeneous enhancement (Figure 2e). The time signal intensity curves revealed a washout pattern for the mass (Figure 2f). The patient underwent mass resection. Macroscopically, the tumour, measuring 2.1 cm × 1.6 cm × 1.1 cm, presented as a solid white elastic nodule with a smooth surface resembling a fibroadenoma. Histological examination of the mass indicated a tubular breast adenoma. The section was greyish-yellow and had an average texture. The lesion was rounded and nodular, surrounded by dense, proliferating glandular ducts with typical epithelial and myoepithelial cell layers (Figure 3a and b).

**Informed consent:** Informed consent has been obtained from all individuals included in this study.
**Ethical approval:** The research related to human use has been complied with all the relevant national regulations, institutional policies and in accordance with the tenets of the Helsinki Declaration, and has been approved by the authors’ institutional review board or equivalent committee.

## 3 Discussion

Tubular adenomas are rare benign tumours of the breast that are often found in young women. Typical clinical manifestations include a palpable, painless mass with clear boundaries, good manoeuvrability, soft texture, and no changes in the surrounding skin or nipples, and the axillary lymph nodes are generally not swollen. Tubular adenomas almost always occur in the normal location of breast tissue, but it has also been reported to occur in accessory breast tissues [6]. Histologically, tubular adenoma is composed of a circumscribed mass of tightly packed tubules surrounded by minimal stroma. The tubules are lined by one layer of epithelial cells and a thin layer of myoepithelial cells [7]. Unlike fibroadenomas, which contain a large amount of fibrous stroma, there is scant stroma surrounding the packed acinar cells [8].

Tubular adenomas and fibroadenomas of the breast have similar clinical presentations and imaging features, and identifying their pathologic types based on imaging is challenging [1]. Ultrasound is the most common breast exam for young women. It has been reported that tubular adenomas of the breast usually appear on ultrasound as well-defined hypoechoic masses with long axes parallel to the skin [9]. MRI is advantageous as it can be performed in multiparametric mode, which can provide more differential diagnostic information. In this case, the patient’s MRI showed an oval shape with a lobulated appearance, clear borders, and an inhomogeneous signal from the mass on enhanced imaging. A rare case of a giant cystic tubular adenoma of the breast was previously reported in which an uneven, internal signal on T1- and T2-weighted images reflected secretory products and proteins that were histologically trapped in the tubular adenoma [10]. It has been suggested that the rich ductal component of tubular adenomas may be the pathologic basis for their heterogeneous signals [8]. The lobulated appearance may be related to the fact that tubular adenomas contain a large proportion of epithelial cells, which are softer in texture, and as the tumour continues to grow, the fibrous tissue septum divides it into multiple nodules [11]. Fibroadenomas have more mesenchymal components, which are harder and less likely to form a lobulated structure. This may be one of the points of differentiation between tubular adenomas and fibroadenomas. Lobular tumours and lactating adenomas may also be elliptical with a lobulated morphology, but lobular tumours are often associated with a fissure-like low signal within the tumour and rapid enlargement of the mass in a short period, allowing them to be differentiated by careful examination [12]. Lactating adenomas are usually associated with pregnancy, breastfeeding, or a history of oral contraceptive use, which may provide a basis for differentiation [13].

Calcifications may be a sign of benign changes, but they can also be a product of malignant processes. In this case, no microcalcifications within the lesion were detected on ultrasound or MRI. A previous study reported tubular adenomas in young women who did not show calcifications during mammography and whose appearance was similar to that of noncalcified fibroadenomas, whereas in older patients, tightly packed punctate or irregular calcifications may be observed [1]. This may be related to the older age of the patients, the longer growth time of the lesion, the blood circulation disorder of the tumour, and the calcium salt deposition that occurs after tissue necrosis [11]. The calcification foci of tubular adenomas usually have certain characteristics, such as a dense, punctate, or irregular shape, and the calcification distribution is dense relative to the lesion volume [5]. Microscopically, these large, round clumps of calcium and phosphorus are located within the dilated acinar glands [8]. The distribution and morphology of malignant calcifications in the breast are usually different from those of tubular adenomas, which are usually characterized by finer particles, larger numbers, and wider distributions [14]. The different characteristics of intralesional calcification can help in distinguishing tubular adenoma from breast cancer. However, more attention should be paid to calcifications because they may be a precursor of malignant processes from a radiological perspective. Currently, biopsy of these lesions is still necessary to exclude a malignant process.

Tubular adenoma of the breast is a disease distinct from fibroadenomas and fibroepithelial lesions, making it necessary to describe its cytologic features. The presence of tubules and stromal debris has been found to be an independent factor associated with tubular adenomas in comparison with fibroadenomas, while the predominance of large epithelial debris and bare stromal debris are independently associated with fibroadenomas. These findings not only reflect the histologic differences between tubular adenomas and fibroadenomas but also confirm that tubular adenomas have cytologically distinguishable features from fibroadenomas and that tubular adenomas can be diagnosed by fine-needle aspiration cytology [15]. Mammary ductal adenomas are pathologically benign tumours that rarely recur. However, some scholars have found that this benign lesion can evolve into intraductal carcinoma *in situ* if it persists for a long time [14], so we believe that it should be taken seriously. Therefore, observing the details of the margins, morphology, and internal signal and the degree of uniformity of enhancement of the mass, combined with the patient’s history, is conducive to differential diagnosis.

## 4 Conclusion

Tubular adenoma of the breast occurs in young women of childbearing age and is a rare benign epithelial lesion. Preoperative diagnosis is difficult because, in most cases, the clinical manifestations and imaging features resemble fibroadenomas. In this case, the internal heterogeneous signals of the mass and the oval shape of the lobulated mass were related to the pathological changes in the tubular adenoma and can play a role in the differential diagnosis of tubular adenoma, helping to improve the preoperative diagnosis rate.
